# Antiglycation and Antioxidant Effect of Nitroxyl towards Hemoglobin

**DOI:** 10.3390/antiox11102007

**Published:** 2022-10-11

**Authors:** Olga V. Kosmachevskaya, Elvira I. Nasybullina, Igor S. Pugachenko, Natalia N. Novikova, Alexey F. Topunov

**Affiliations:** 1 Bach Institute of Biochemistry, Research Center of Biotechnology of the Russian Academy of Sciences, 119071 Moscow, Russia; 2 National Research Center “Kurchatov Institute”, 123182 Moscow, Russia

**Keywords:** hemoglobin, nitroxyl, Angeli’s salt, methylglyoxal, advanced glycation end products, antiglycation and antioxidant effect

## Abstract

Donors of nitroxyl and nitroxyl anion (HNO/NO^−^) are considered to be promising pharmacological treatments with a wide range of applications. Remarkable chemical properties allow nitroxyl to function as a classic antioxidant. We assume that HNO/NO^−^ can level down the non-enzymatic glycation of biomolecules. Since erythrocyte hemoglobin (Hb) is highly susceptible to non-enzymatic glycation, we studied the effect of a nitroxyl donor, Angeli’s salt, on Hb modification with methylglyoxal (MG) and organic peroxide―*tert*-butyl hydroperoxide (*t*-BOOH). Nitroxyl dose-dependently decreased the amount of protein carbonyls and advanced glycation end products (AGEs) that were formed in the case of Hb incubation with MG. Likewise, nitroxyl effectively protected Hb against oxidative modification with *t*-BOOH. It slowed down the destruction of heme, formation of carbonyl derivatives and inter-subunit cross-linking. The protective effect of nitroxyl on Hb in this system is primarily associated with nitrosylation of oxidized Hb and reduction of its ferryl form, which lowers the yield of free radical products. We suppose that the dual (antioxidant and antiglycation) effect of nitroxyl makes its application possible as part of an additional treatment strategy for oxidative and carbonyl stress-associated diseases.

## 1. Introduction

Diabetic hyperglycemia is defined by the increased concentration of both glucose and the products of its metabolism—methylglyoxal (MG) and glyoxal—in the organism [1]. These aldehydes are extremely reactive compounds that spontaneously react with amine, guanidine and thiol groups of long-lived proteins and other macromolecules. This non-enzymatic interaction triggers formation of advanced glycation end products (AGEs), followed by intra- and intermolecular crosslinking [1]. It deteriorates the properties of proteins and other biomolecules. Moreover, this modification is irreversible since there are no enzymes in the organism capable of hydrolyzing AGEs. The protein glycation process can lead to formation of certain types of globular protein aggregates or amyloid fibrils accumulated in cells [2,3].

A high level of AGEs leads to many pathological conditions. Among the sequelae of hyperglycemia are nephropathies, microangiopathies, atherosclerosis, retinopathy, degeneration of the eye lens and neurodegenerative diseases. For instance, Advanced glycation end products contribute to vascular disease development [4]. The AGEs form cross-linking of extracellular matrix proteins, which leads to blood vessel wall stiffness. In addition, AGEs are bound to specific cellular receptors (receptors for advanced glycation end products, RAGEs) that trigger the generation of pro-inflammatory and pro-fibrotic cytokines and chemokines, as well as reactive oxygen species (ROS) [4,5]. In general, these phenomena promote the development of arterial hypertension, atherosclerosis, hemolytic anemia, vascular occlusion, local ischemia and formation of hypercoagulability [5,6].

A situation when excessive concentration of sugars and reactive carbonyl compounds (glyoxal and MG) leads to an increase in AGE formation rate was named as “carbonyl stress” [7]. Notably, AGE formation is often associated with oxidative stress [7,8,9,10,11,12]. Free radicals, including ROS, are formed in reactions with MG. At the same time, ROS themselves produce new AGEs forming a complex yet vicious circle. To reduce the harmful effects of hyperglycemia, inhibitors of AGE formation are used. The ideal inhibitor has to combine the properties of a “trap” for reactive carbonyl compounds, of an antioxidant and a chelator of redox-active metal ions (copper and iron), which catalyze auto-oxidation of glucose.

The nitroxyl molecule (HNO/NO^−^) has a set of remarkable properties that make it a promising inhibitor of AGE formation or a putative anti-glycation agent. HNO is formed from NO by one-electron reduction and protonation [13], and subsists in an equilibrium with nitroxyl anion (NO^−^) [14]. However, nitroxyl mainly exists in the HNO form, since it has higher stability than NO^−^ under physiological conditions [13,15]. HNO has a high biological activity, largely overlapping with the effect of nitric oxide (NO) or peroxynitrite (ONOO^−^) [16].

Grounded in its strong electrophilic properties, HNO easily reacts with nucleophilic protein groups (for example, SH− ones), blocking them [17,18]. Being an effective electron and proton donor, HNO can also act as a classic antioxidant [19]. In addition, this NO metabolite can be involved in forming dinitrosyl iron complexes (DNICs); these are effective antioxidants and reduce the toxic effect of iron under oxidative stress conditions [20,21,22].

**Figure 1:**
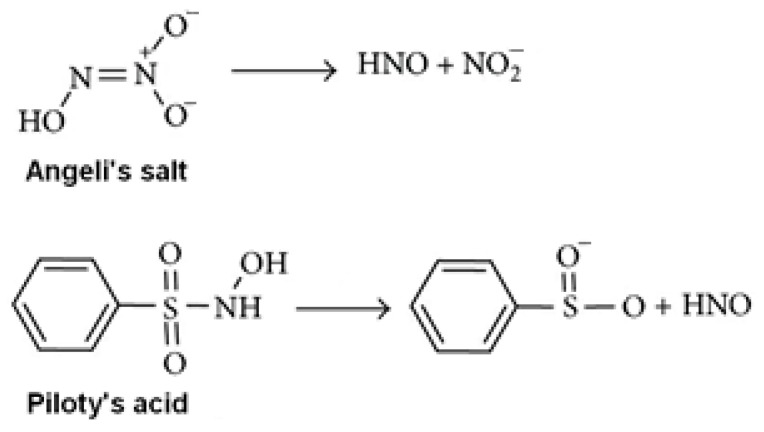
Nitroxyl donors, Angeli’s salt and Piloty’s acid, and ways of their destruction and release of nitroxyl (HNO).

HNO is a short-lived compound due to a rapid dimerization into hypo–nitrous acid that further decomposes into N_2_O and H_2_O (the rate constant of *k* = 8 × 10^6^ M^−1^s^−1^) [15]. Therefore, HNO donor molecules are used in biomedical research and pharmacology. These donors decompose and produce HNO in response to various external stimuli, such as pH, temperature or light [23,24]. HNO donors are usually hydroxylamine derivatives with good leaving groups attached to the nitrogen atom, and nitroso compounds (X–N=O, where X is the leaving group) [23]. The most commonly used HNO donors are Angeli’s salt and Piloty’s acid (Figure 1). Angeli’s salt, sodium trioxodinitrate, is a sodium salt of nitrohydroxamic acid. Piloty’s acid, benzenesulfohydroxamic acid, is an organic compound, the benzenesulfonyl derivative of hydroxylamine.

The antiglycation effect of HNO may be associated with NO, which, as was shown by Asahi et al. [25], slows down the formation of fluorescent AGEs. We showed that one HNO donor, Piloty’s acid, decreased the toxic effect of MG on bacterial culture, increasing cell viability and decreasing the autofluorescence of protein-bound non-enzymatic glycation products [26].

Taking into account that there are practically no data on HNO’s effect on protein glycation by MG, we set out to study this effect on hemoglobin (Hb) modification induced by oxidation and glycation. Erythrocytic Hb was chosen due to its high susceptibility to non-enzymatic glycation. Moreover, the lifetime of Hb is long enough to react with glucose or MG [27]. Angeli’s salt was used in our research, since it was reported to be an effective HNO donor [23]. It easily decomposes (t_1/2_ = 3 min) at physiological pH and temperature with the subsequent formation of HNO and NaNO_2_ [13,28]. HNO has the ionization constant (*pK_a_*) = 11.4. Therefore, at physiological pH it exists mainly in a protonated form [15].

## 2. Materials and Methods

### 2.1. Materials

The following reagents were used in the present work: 5-amino-2,3-dihydrophthalazine-1,4-dione (luminol), aminoguanidine, 2-amino-2-(hydroxymethyl)-1,3-propanediol (Tris), bovine Hb (is sold in oxidized form, metHb, Hb-Fe^III^), bromophenol blue, catalase from bovine liver, Coomassie brilliant blue R-250, 2′,7′-dichlorodihydrofluorescein (DCFH), 2,4-dinitrophenylhydrazine (DNPH), 2-diethoxyphosphoryl-2-methyl-3,4-dihydro-2H-pyrrole-l-oxide (DEPMPO), N-ethylmaleimide (NEM), glycine, glycerol, methylglyoxal (MG), polyacrylamide (PAA), pyridine, sodium azide (NaN_3_), sodium dithionite, sodium dodecyl sulfate (SDS), *tert*-butyl hydroperoxide (*t*-BOOH), *N*^1^,*N*^1^,*N*^4^,*N*^4^-tetramethylbenzene-1,4-diamine (TMPD), trichloroacetic acid (TCA), all — “Sigma-Aldrich” (St. Louis, MO, USA); HNO donor Sodium trioxidinitrate (Angeli’s salt), “Cayman Europe” (Tallinn, Estonia); and 5-(diethoxyphosphoryl)-5-methyl-1-pyrroline-N-oxide (DEPMPO), “Oxis” (Portland, OR, USA).

The Hb modified with NaN_3_ and NEM was also used in the work. For obtaining these forms, 0.6 mM NaN_3_ or 80 mM NEM was added to 0.15 mM metHb in 50 mM PBS (pH 7.4) to final concentrations 55 mM and 0.4 mM, respectively.

Different concentrations of the various substances were used to make the effect of their action more clearly visible.

### 2.2. Hb Modification with MG. Studying the Effect of Angeli’s Salt and Aminoguanidine

Non-enzymatically glycated Hb was obtained in vitro during Hb incubation with MG for 1–4 days at 37 °C. Methylglyoxal solution (40%) was added to 0.15 mM Hb (metHb-H_2_O or metHb-N_3_) solution in 50 mM PBS (pH 7.4) to achieve 54 mM final concentration (molar ratio 1:90 per Hb subunit). The MG excess was removed by dialysis in 20 mM sodium phosphate buffer (PBS) with pH 7.4 overnight at 4 °C. Sampling for AGE analysis was carried out every 24 h.

To study the antiglycation effect of Angeli’s salt and aminoguanidine, the reaction mixture was incubated for 20 min, then Angeli’s salt in 10 mM NaOH or water aminoguanidine solution was added to various final concentrations of reagents and incubated for 24 h at 37 °C.

### 2.3. Measurements of Hb-Bound AGEs

Hb glycation degree was analyzed based on the fluorescence of protein-associated AGEs (λ_ex_ = 334 nm, λ_em_ = 440 nm). Protein samples were diluted in a ratio of 1 to 10 with 20 mM PBS (pH 7.4) prior to all measurements. Fluorescence was recorded using a Shimadzu RF-5301PC fluorescence spectrophotometer (“Shimadzu”, Kyoto, Japan) in 0.5 mL micro cuvette at a medium scanning speed, with high sensitivity (according to the unit designation), and a slit width of excitation and emitting light of 5 nm and 10 nm, respectively.

### 2.4. SDS-Electrophoresis

Electrophoresis was performed in 12% PAAG blocks with a size of 15 cm × 15 cm × 1 mm according to the Laemmli method [29] with a VE-series vertical electrophoresis device (“Helicon”, Moscow, Russia). MetHb samples, prepared as described above, were diluted with the sample buffer in the proportion 1:1, and heated at 95 °C for 5 min. The sample buffer contained 0.1 M Tris-HCl (pH 6.8), 4% SDS, 0.2% bromophenol blue and 20% glycerol. A 3% DTT solution was added to the buffer solution to ensure conditions for reduction; 10 µL of the sample was loaded to the gel. The electrode buffer contained 0.2 M Tris-glycine (pH 8.3), and 0.1% SDS. Electrophoresis was carried out at 4 °C, I = 50 mA, and U = 150 V. “Elf-4” (“DNA-Technology”, Moscow, Russia) was used as a power source for electrophoresis. After completing protein separation, the gel was fixed and stained with Coomassie brilliant blue R-250 solution.

### 2.5. Gel Chromatography

Hb samples (prepared as described in Section 2.2) were purified on Toyopearl HW-55F (“Toyo Soda”, Tokyo, Japan) column (43 cm × 2.2 cm) with 25 mM Tris-HCl buffer (pH 7.5) and 0.2 M NaCl. Column flow rate of 20 mL/hour was maintained using 2132 Microperpex Peristaltic Pump (“Pharmacia LKB Biotechnology”, Uppsala, Sweden). The volume of Hb solution used in the column was 1.5 mL. The outlet of the fractions was registered with the optical density at 408 nm. All procedures were carried out at 4 °C.

### 2.6. Oxidative Modification of Hemoglobin by tert-Butyl Hydroperoxide

To study the HNO effect on Hb oxidative modification, *t*-BOOH was used as an oxidant at different final concentrations: 0.4, 0.6, 1.0, 1.6, 2.6, 4.2 mM. *t*-BOOH was added to 0.15 mM metHb-H_2_O solution in 50 mM PBS (pH = 7.4). A solution of Angeli’s salt in 10 mM NaOH was added to provide for the final concentration of 2 mM. After 15 min of Hb incubation, the content of carbonyl derivatives and the number of intact heme groups were determined.

### 2.7. Measurements of Protein Carbonyls

Carbonyl Hb derivatives were quantified using the method [30] with minor modifications. The method consists in inducing formation of covalent adducts of carbonyl (aldo- and keto-) groups with DNPH, spectrophotometrically recorded at 13 wavelengths. The amount of formed 2,4-dinitrophenylhydrazones was calculated using the formula:

S = S_1_ + S_2_,

where

S_1_ = (E_230_ + E_254_) × 12 + (E_254_ + E_270_) × 8 + (E_270_ + E_280_) × 5 + (E_280_ + E_356_) × 38 + (E_356_+ E_363_) × 3.5 + (E_428_ + E_520_) × 46,

and

S_2_ = (E_363_ + E_370_) × 3.5 + (E_370_ + E_428_) × 29 + (E_430_ + E_434_) × 2 + (E_520_ + E_535_) × 7.5.

S_1_, aldehyde-dinitrophenyl hydrazones; S_2_, neutral ketone-dinitrophenyl hydrazones.

The samples were prepared in the following way: 0.5 mL of DNPH solution in 2 M HCl was added to 0.1 mL of Hb solution and incubated for 1 h. Protein precipitation was induced through adding 0.5 mL of 20% TCA. After 10 min, the samples were centrifugated at 3000× *g* for 15 min. To remove unbound DNPH, the protein precipitate was thrice washed with 0.4 mL of ethanol and ethyl acetate mixture (1:1). The obtained precipitate was air-dried and dissolved in 1 mL of 8 M urea solution. The samples were five-fold diluted prior to measurements.

### 2.8. Measurements of the Heme Group

Heme concentration in metHb solution was measured using the modified pyridine hemochrome method. One hundred and thirty-five µL of water and 450 µL of 30% pyridine alkaline solution were added to 15 µL of metHb sample prepared as described in Section 2.2. Before measurement, the solution was reduced with sodium dithionite. Optical absorption of the reduced heme complex with pyridine was measured at 556 and 539 nm. The heme concentration was calculated using the difference A_556_–A_539_, considering that (E_556–539_ = 4.3 mM^−1^ cm^−1^)

### 2.9. Evaluation of Antioxidant/Antiradical Properties of Nitroxyl

#### 2.9.1. Luminol-Dependent Chemiluminescence

Formation of free radical intermediates in Hb reaction with MG and *t*-BOOH was evaluated by chemiluminescence with luminol as an activator. When luminol interacts with oxidizing agents (ROS), it emits a blue glow, prior to returning from excited to normal electronic state.

In Hb reaction with MG, the reaction mixture contains 0.15 mM MetHb-H_2_O in 50 mM PBS (pH 7.4), 54 mM MG (molar ratio 1:90 per Hb subunit), and 8 mM Angeli’s salt. The mixture was incubated for 24 h at 37 °C. Before measurement, luminol was added to achieve 2 mM final concentration. When Hb reaction with *t*-BOOH was studied, the composition of the reaction mixture was as follows: 0.15 mM MetHb-H_2_O in 100 mM PBS (pH 7.4), 0.6 mM *t*-BOOH and 2 mM luminol.

Chemiluminescence was measured for 6 min using the chemiluminescence analyzer Lum-5773 (“DISoft”, Moscow, Russia). Registration started 2–3 s after mixing the reaction mixture components. The data are presented as the area under the chemiluminescence curve (AUC) calculated for each sample.

#### 2.9.2. Obtaining and Registration of Oxoferrylhemoglobin

OxoferrylHb (Hb-Fe^IV^ = O) was obtained by adding 0.4 mm H_2_O_2_ to 0.03 mM metHb in 50 mM PBS (pH 7.4) in the proportion 1:3 per Hb subunit. Catalase (EC 1.11.1.6) was added to the final dose 365 units/mL after 3 min of incubation to remove the unreacted peroxide. After that, Angeli’s salt was added to Hb-Fe^IV^ = O solution in the proportion 1:1 per Hb subunit to final 0.12 mM concentration. Reduction of Hb-Fe^IV^ = O to metHb was studied based on the absorption spectra in the 450–700 nm range. There was a decrease in absorption at 541 nm and 582 nm and an increase at 631 nm.

The effect of Angeli’s salt on Hb-Fe^IV^ = O formation was also studied in the system with *t*-BOOH. In this case, 0.71 mM *t*-BOOH was added to 0.03 mM Hb in 50 mM PBS (pH 7.4) in the proportion 1:6 (per Hb subunit). Angeli’s salt was added simultaneously with *t*-BOOH in 0.4 mM concentration, which corresponds to the proportion 1:3 per Hb subunit. Hb-Fe^IV^ = O accumulation was monitored based on the absorption difference at 582 nm and 617 nm (the nearest isosbestic point).

#### 2.9.3. TMPD Oxidation

The ROS production in MetHb-H_2_O reaction with *t*-BOOH was measured in the presence of TMPD using the method described by Feng et al. [31]. *t*-BOOH was added to 0.03 mM Hb solution in PBS (50 mM, pH = 7.4) with 0.12 mM TMPD. The final *t*-BOOH concentration was 1.42 mM. The optical absorption was measured at 610 nm. Angeli’s salt was added to the reaction mixture to achieve final concentrations of 0.2, 0.4, 0.6 and 0.8 mM. The area under TMPD oxidation curve, registered within 5 min, was applied as the indicator for assessing oxidizer generation level.

#### 2.9.4. Detecting ROS with a Fluorescent Probe

The ROS amount was also measured using DCFH fluorescent probe. In DCFH reaction with ROS, a reactive intermediate is formed, which, after interaction with molecular oxygen, produces a fluorescent compound, 2′,7′-dichlorodihydrofluorescein (DCF), with higher stability, and superoxide [32]. The DCF fluoresces at λ_ex_ = 485–500 nm and λ_em_ = 515–530 nm. The reaction mixture contained 0.03 mM MetHb-H_2_O, 0.6 mM DCFH and 0.71 mM *t*-BOOH in 100 mM PBS (pH 7.4). The mixture was incubated at 20 °C for 5 min in the presence and in the absence of Angeli’s salt. The measurements were carried out using Shimadzu RF-5301PC fluorescence spectrophotometer in 0.5 mL micro cuvette at a low scanning speed, with high sensitivity (according to the unit designation), and 3 nm slit width of both excitation and emitting light.

#### 2.9.5. UV-Visible Spectrophotometry

Optical absorption spectra in all cases were recorded using the UV-VIS spectrophotometer Hitachi U-2910 (“Hitachi”, Tokyo, Japan) at 20 °C in a 1 cm optical glass cuvette at a scanning speed of 600 nm/min.

### 2.10. Statistical Analysis

All experiments were carried out in at least three replications. The data are presented as a mean ± standard deviation. Student *t*-test was used for analysis of data obtained.

## 3. Results

### 3.1. Effect of Nitroxyl on Non-Enzymatic Hb Glycation

#### 3.1.1. Spectrofluorescence Analysis of AGE Formation

Some AGEs (pentosidine, argpyrimidine, pyrrolopyridine, pentodilysine, vesperlysines etc.) show autofluorescence [32,33,34,35], and the process of AGE formation on proteins can be registered by the increase in fluorescence at certain wavelength (λ_ex_ = 334 nm, λ_em_ = 440 nm) [36].

The antiglycation effect of HNO was studied using the carbonyl stress modeling experimental system. Hb of bovine erythrocytes was the object of this study. MG was used as a glycating agent. Its high level is found in erythrocytes upon diabetic hyperglycemia [27,37]. The antiglycation activity of HNO was compared with that of aminoguanidine, which is known as an effective AGE scavenger [38,39], and was used as standard antiglycating agent (positive control).

**Figure 2:**
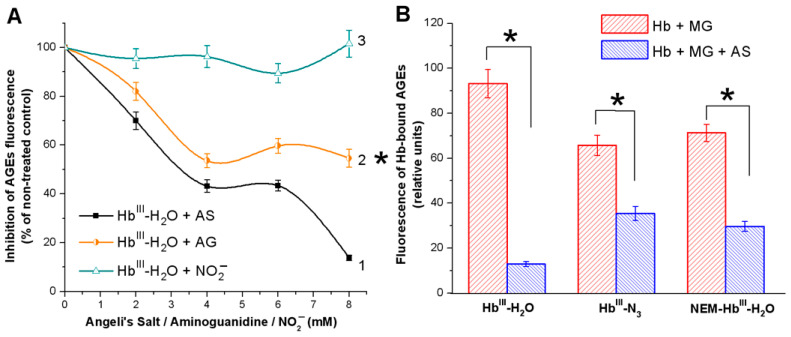
Formation of fluorescent advanced glycation end products (AGEs) in hemoglobin (Hb) reaction with methylglyoxal (MG). (**A**) effect of Angeli’s salt—1, of aminoguanidine—2, and of nitrite ions (NO_2_^−^)—3; (**B**) effect of Angeli’s salt (8 mM) on Hb modified with NaN_3_ and NEM. Reaction mixture in (**A**,**B**): 0.15 mM of oxidized hemoglobin (metHb) in 50 mM PBS (pH 7.4), 54 mM MG. Results are depicted as the mean with S.E.M. of three independent experiments (*n* = 3). Two-way ANOVA (**A**) (* *p* ≤ 0.05), student *t*-test (**B**) (* *p* ≤ 0.05).

Both Angeli’s salt and aminoguanidine inhibited the formation of fluorescent Hb-bound AGEs depending on the dose (Figure 2A, Curves 1 and 2), and the inhibitory effect of Angeli’s salt was more evident. Since, during the decomposition of Angeli’s salt, one mole of HNO and one mole of NO_2_^−^ are produced, the effect of the same concentration of NO_2_^−^ on AGE formation was studied (Figure 2A, Curve 3). Nitrite ions had no effect on Hb glycation by MG, which indicates that the observed antiglycation effect of Angeli’s salt is due to HNO action. HNO can also affect Hb thiols and heme group [18,40]. Therefore, it can be assumed that the antiglycation effect of HNO is due to its antioxidant properties.

The mechanism of aminoguanidine antiglycation effect is different. It has strong nucleophilic properties, and forms adducts with α-dicarbonyl compounds (MG, glyoxal, 3-deoxyglucosone), with production of triazines blocking the conversion of Amadori products into AGEs [34]. Aminoguanidine can also act as an antioxidant and chelating agent, suppressing the formation of hydroxyl radicals and lipid peroxidation (LPO) in cells and tissues [41].

Therefore, a similar study was carried out with Hb^III^-N_3_ and NEM-Hb^III^-H_2_O. The formation of a heme complex with N_3_^−^ inhibited AGE formation by 30%. At the same time, blocking of reactive SH groups of Hb (Cysß93) with NEM (reactive towards thiols, commonly used as a modifier of protein cysteine residues) inhibited AGE formation by 24% (Figure 2B). This indicates that heme iron and SH groups are involved in Hb modification by MG. Moreover, in the latest variants, the antiglycation effect of Angeli’s salt was less pronounced, compared with Hb not treated with NaN_3_ and NEM.

It is known that NO can interact with non-enzymatic glycation products [25,42]. At the same time, various NO donors inhibit pentosidine formation [25]. This is related to NO ability to intercept free radicals, which emerge as a result of non-enzymatic glycation reactions [43,44].

#### 3.1.2. Analysis of the Formation of Cross-Linked Hb

**Figure 3:**
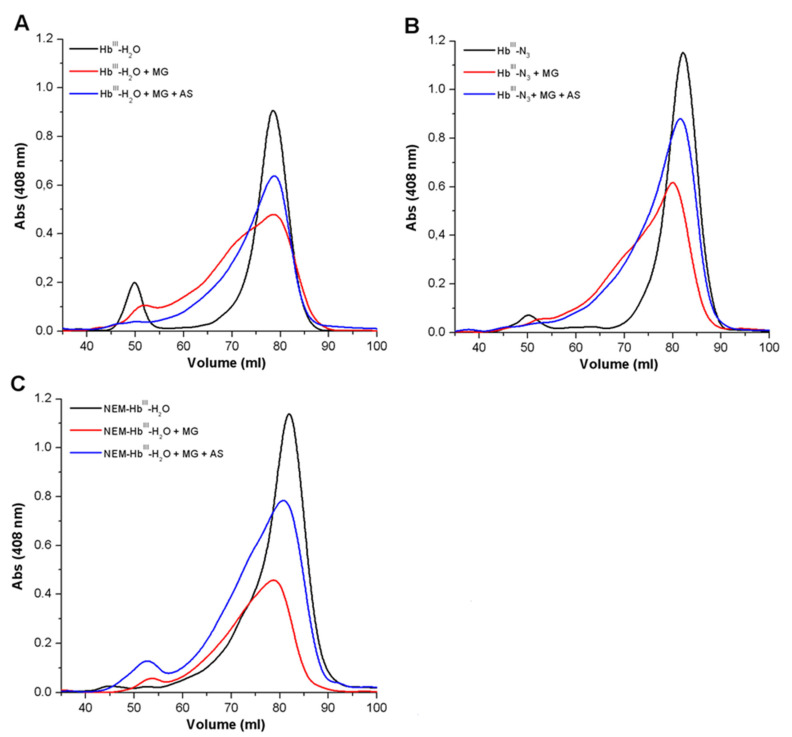
Hb elution profiles from the Toyopearl HW-55F column. (**A**) metHb-H_2_O; (**B**) metHb-N_3_; (**C**) NEM-metHb-H_2_O. The control (without MG), black curves; with MG (13.5 mM), red curves; with MG (13.5 mM) + Angeli’s salt (8 mM), blue curves. Reaction mixtures, as in Figure 2.

Since AGEs produced as a result of non-enzymatic glycation form inter-protein bonds, we studied the effect of Angeli’s salt on the formation of cross-linked Hb. Gel filtration chromatography on Toyopearl HW-75F column (Toyo Soda, Tokyo, Japan) was used. The elution profile of intact Hb (metHb-H_2_O) had two clear peaks at 50 mL and 87 mL (Figure 3A, black curve), which correspond to dimeric and monomeric Hb forms, respectively. Peak I (50 mL) was the minor fraction. In the case of Hb^III^-N_3,_ it was even smaller (Figure 3B, black curve), and in the case of NEM-Hb^III^-H_2_O it was almost imperceptible (Figure 3C, black curve). This can be explained by the influence of available (not N_3_^—^ or NEM-modified) heme and SH group on auto-oxidation processes in experimental procedures.

Gel filtration chromatography was also used for Hb samples previously incubated with MG, or with MG combined with Angeli’s salt (8 mM) for 24 h. Incubation with MG resulted in decrease in metHb-H_2_O monomeric fraction and an increase in fractions between the first and second peaks (Figure 3A, red curve). This indicates that glycated Hb molecules and subunit cross-linking are formed. MG-induced modification also led to forming large Hb aggregates, which did not penetrate the column and settled on the surface layer. In the case of Hb^III^-N_3_, the share of such aggregates was 15%, and in the case of NEM-Hb^III^-H_2_O it was ~60% of the total Hb sample. These data show intensive modification of Hb molecules by glycation products, with the subsequent formation of inter-subunit crosslinking.

Angeli’s salt had a protective effect on Hb regardless of the protein form (Figure 2, blue curves). At the same time, it leveled down not only the amount of AGE-encrusted Hb molecules, but also the amount of cross-linked (multimeric) Hb molecules. In the presence of Angeli’s salt, Hb oligomerization practically did not occur. This is consistent with the results shown in Figure 2.

**Figure 4:**
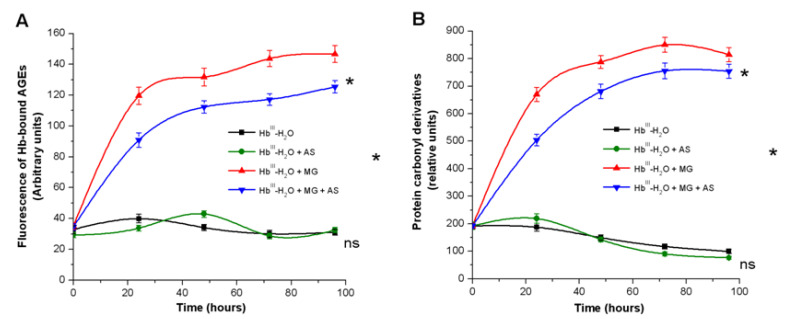
The effect of Angeli’s salt on formation of fluorescent AGEs (**A**) and carbonyl groups (**B**) in metHb-H_2_O reaction with MG. Reaction mixture: 0.15 mM metHb-H_2_O in 50 mM PBS (pH 7.4), 54 mM MG, 3.2 mM Angeli’s salt. Results are depicted as the mean with S.E.M. of three independent experiments (*n* = 3). Two-way ANOVA (**A**,**B**) (* *p* ≤ 0.05; ns: non-significant).

During the next stage, we studied the effect of Angeli’s salt on Hb modification by MG in dynamics. For these experiments, 3.2 mM of Angeli’s salt was selected, which corresponded to the half-inhibition concentration (IC_50_). Adding Angeli’s salt to the reaction mixture containing MetHb-H_2_O decreased the level of fluorescent AGEs and protein carbonyls by ~30% after 24 h (Figure 4A). The inhibitory effect persisted throughout the incubation time (96 h).

### 3.2. Effect of Nitroxyl on Hb Oxidative Modification in Reaction with t-BOOH

It is known that Hb glycation by glucose, fructose and MG leads to decomposition of the heme group as a result of endogenous ROS formation [10,45]. Therefore, we decided to study the effect of Angeli’s salt on Hb oxidative modification in the reaction with *t*-BOOH.

**Figure 5:**
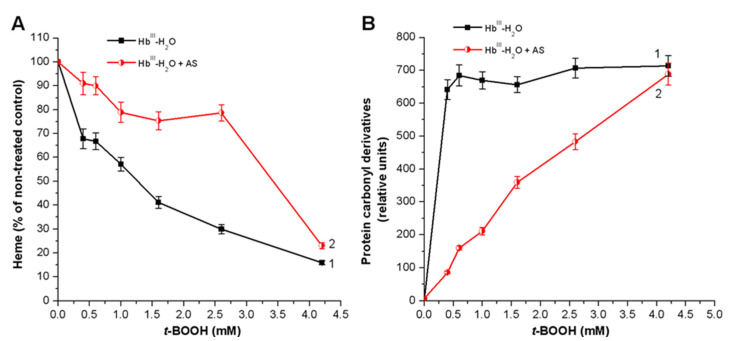
Effect of Angeli’s salt on heme group destruction (**A**) and formation of carbonyl groups on metHb-H_2_O; (**B**) under the influence of various *t*-BOOH concentrations. Control, without Angeli’s salt (1), with Angeli’s salt (2). Reaction mixture: 0.15 mM metHb-H_2_O in 100 mM PBS (pH 7.4), 2 mM Angeli’s salt. Results are depicted as the mean with S.E.M. of three independent experiments (*n* = 3). Student *t*-test (**A**) (*p* ≤ 0.05), student *t*-test (**B**) (*p* ≤ 0.05 for all point except 4.25 mM *t*-BOOH, for this point, non-significant).

Under the influence of various *t*-BOOH concentrations, the Hb heme group was destroyed (Figure 5A, black curve). Adding Angeli’s salt to the reaction mixture exerted a significant protective effect on heme (Figure 5A, red curve). In addition, in this experimental system, the HNO donor, Angeli’s salt, prevented the formation of new protein carbonyl groups (Figure 5B, red curve) that usually result from oxidative damage of Hb amino acid residues. The protective effect of Angeli’s salt is particularly noticeable at low concentrations of *t*-BOOH.

**Figure 6:**
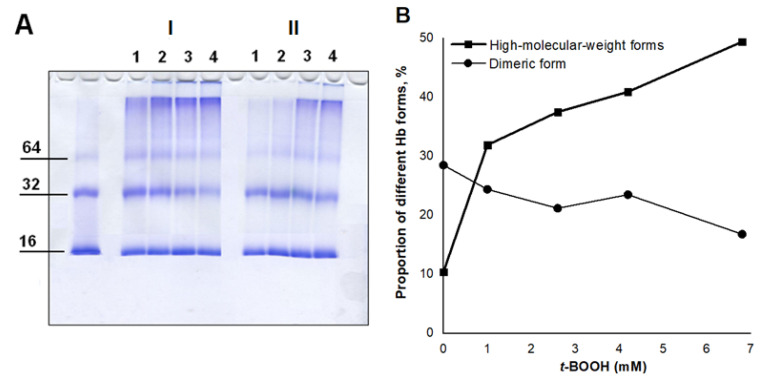

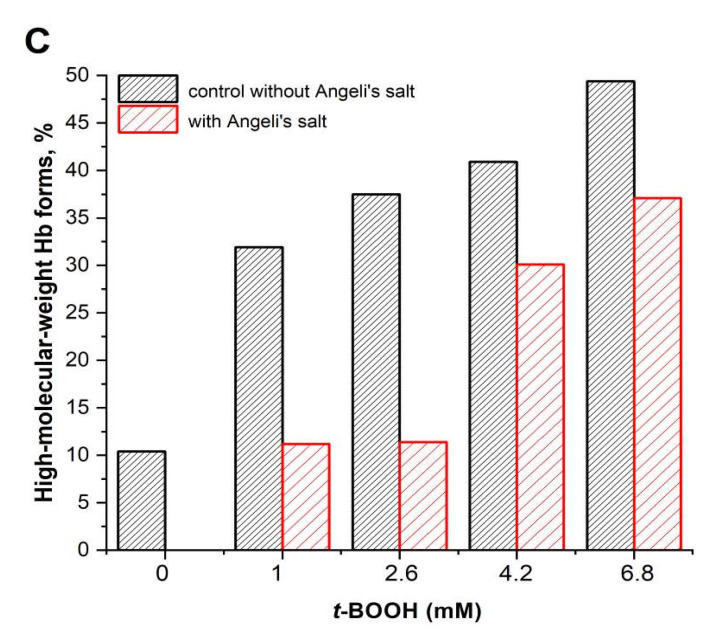
Effect of Angeli’s salt on Hb (MetHb-H_2_O) subunit aggregation under the influence of *t*-BOOH: (**A**) SDS-electrophoresis in 12% polyacrylamide gel. I, control without Angeli’s salt; II, with Angeli’s salt (2 mM). The numbers indicate *t*-BOOH concentration variants: 1–1 mM, 2–2.6 mM, 3–4.2 mM, 4–6.8 mM. Preparation of protein samples as shown in Figure 5; (**B**) Portions (%) of high-molecular (over 64 kDa) and dimeric (32 kDa) Hb forms under the action of various *t*-BOOH concentrations; (**C**) Portions (%) of high-molecular (over 64 kDa) Hb form under *t*-BOOH action with and without Angeli’s salt. The data presented in panels B,C were obtained by processing the electrophoresis image using the Image Lab Software, Bio-Rad program. Figure shows the result of typical electrophoresis experiment. Other electrophoresis experiments yielded similar results.

This was also confirmed with SDS-electrophoresis in 12% polyacrylamide gel (Figure 6). The leftmost band is the control one (Hb without additives), which is represented mainly by monomeric (16 kDa) and dimeric (32 kDa) Hb forms. When oxidant concentration increased, the amount of high-molecular-weight Hb forms increased as well. The use of different *t*-BOOH concentrations made the effect of its action especially visual. In the case of high *t*-BOOH concentrations (4.2 and 6.8 mM), protein aggregates were so large that they could not penetrate the concentrating gel (Figure 6A, I, tracks 3 and 4). That resembled the situation with column chromatography of glycated Hb (Section 3.1).

### 3.3. Effect of Nitroxyl on Free Radical Products Formation in Hb Reactions with MG and t-BOOH

#### 3.3.1. Luminol-Dependent Chemiluminescence

**Figure 7:**
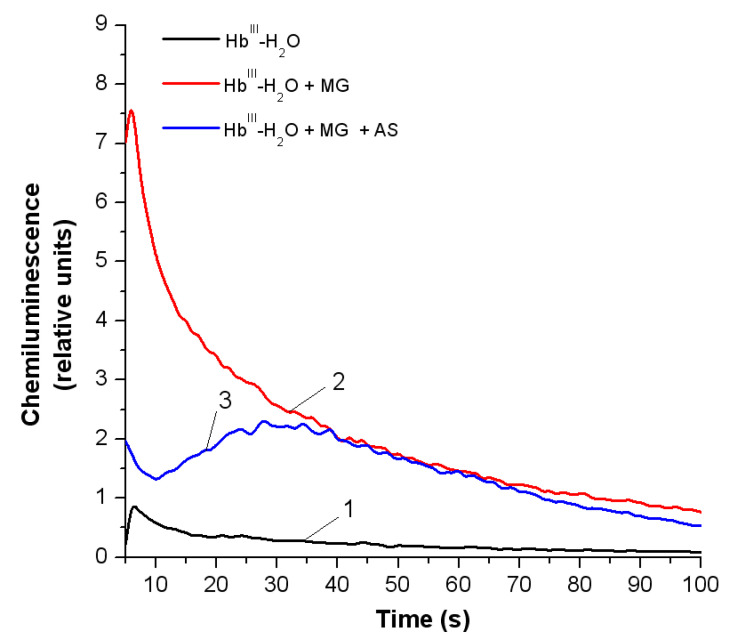
Effect of Angeli’s salt on the formation of free radical products in metHb-H_2_O reaction with MG, measured with luminol-dependent chemiluminescence. Control variant (without additives), black curve (1), with MG, red curve (2), with MG (13.5 mM) combined with Angeli’s salt (8 mM), blue curve (3). Reaction mixture as in Figure 2.

Reactions of amino acids or proteins with MG are accompanied by production of free radicals and ROS (MG radical anion, dialkilimine radical cation, superoxide anion radical) [43,44]. Using luminol-dependent chemiluminescence, we estimated the level of free radical products in the reaction system of metHb-H_2_O incubated with MG (13.5 mM), and with MG (13.5 mM) combined with Angeli’s salt (8 mM) for 24 h (Section 3.1). Chemiluminescence in the mixture was induced by luminol. Figure 7 shows the curves recorded 2–3 s after Angeli’s salt was added. It significantly decreased the amount of free radical reaction products (compare curves 2 and 3).

Free radicals formed during the reaction of MG with protein amino acid residues can be a source of H_2_O_2_ and organic peroxides (in particular lipid and protein hydroperoxides). Hb reactions with organic hydroperoxides are usually accompanied by free radicals of these hydroperoxides and proteins being formed.

**Figure 8:**
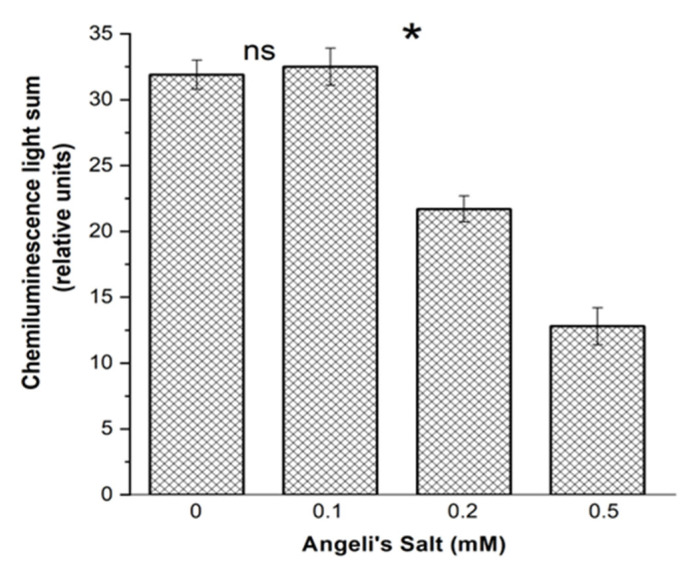
Effect of Angeli’s salt on the formation of free radical intermediates in the reaction of metHb-H_2_O with *t*-BOOH, measured with luminol-dependent chemiluminescence. Reaction mixture: 0.15 mM Hb, 2 mM luminol, 0.6 mM *t*-BOOH in 50 mM PBS (pH 7.4). Preparation of protein samples as in Figure 5. Results are depicted as the mean with S.E.M. of three independent experiments (*n* = 3). One-way ANOVA (* *p* ≤ 0.05; ns: non-significant).

The free radical products in metHb reaction with *t*-BOOH were registered with luminol-dependent chemiluminescence. Angeli’s salt dose-dependently reduced the amount of produced free radicals (Figure 8).

MetHb interaction with peroxides leads to two-electron oxidation of Hb, forming an oxoferryl intermediate with a cation radical on the porphyrin ring (porphyrin^+^–Fe^IV^ = O) [46,47]. This radical further migrates to an amino acid (tyrosine, tryptophan, histidine or cysteine) located near a heme pocket [46,47]. This radical is most frequently located on a tyrosine residue [48].

#### 3.3.2. Peroxyl Radicals’ Formation and Reduction of OxoferrylHb

In cases of an elevated concentration of organic hydroperoxides, they can reduce oxoferrylHb (Hb-Fe^IV^ = O). This reaction is accompanied by peroxyl radical formation (Reaction (1)):

Hb-Fe^IV^ = O + ROOH → Hb^III^ + ROO^•^ (1)

Peroxyl radicals can also be formed on amino acid residues of the protein itself (most often on tryptophan, tyrosine and cysteine ones), when molecular oxygen reacts with the initial amino acid radical [46] (Reaction (2)):

O_2_

porphyrin^+•^-Fe^IV^ = O..Tyr → porphyrin-Fe^IV^ = O..Tyr-O^•^ → porphyrin-Fe^IV^ = O..Tyr^•^-OO^•^ + H_2_O (2)

Intermolecular electron transfer from amino acid radicals may result in intermolecular and intramolecular crosslinking, accompanied by the formation of protein oligomers or heme attaching to the protein.

Therefore, metHb reaction with organic peroxide leads to oxoferrylHb formation with a radical on porphyrin or on amino acid residue (most often on tyrosine, tryptophan, less frequently on cysteine). This induces free radical oxidation of membrane lipids, resulting in alkoxyl (RO^•^) and alkylperoxyl (ROO^•^) radicals being formed as well as inducing lipid hydroperoxide formation (ROOH) (Reactions (3)–(6)) [45,49,50,51]:

Hb-Fe^III^ + ROOH → Hb-Fe^IV^= O + RO^•^ + H^+^ (3)

Hb-Fe^III^ + ROOH → Hb^•^-Fe^IV^= O + ROH (4)

Hb^•^-Fe^IV^= O + ROOH → Hb-Fe^IV^= O + ROO^•^ + H^+^ (5)

Hb-Fe^IV^= O + ROOH → Hb-Fe^III^-OH + ROO^•^ (6)

According to Karoui et al. [52], the main free radical formed in this reaction system is the alkoxy radical (*tert*-butoxy, *t*-BO^•^).

Oxidative Hb modification takes place under the action of these free radical products, leading to a heme decomposition and release of iron ions, catalyzing free radical formation in the Fenton and Haber–Weiss reactions [50].

HNO can neutralize peroxide radicals, since it is an effective donor of a hydrogen atom (Reactions (7) and (8)) [19,28]:

RO^•^ + HNO → ROH + NO^•^ (7)

ROO^•^ + HNO → ROOH + NO^•^ (8)

NO formed as a result of HNO oxidation can also stop free radical lipid oxidation since it interacts with hydroperoxide radicals [53,54,55,56]. NO can effectively produce reactions with ROO^•^ and RO^•^ with the rate constant of *k* = 2 × 10^9^ M^−1^s^−1^ [54]. It was demonstrated that the radical–radical reaction between NO and organic peroxyl radicals is almost a diffusion-limited one, and it generates a transient ROONO species (Reaction (9)):

ROO^•^ + NO^•^ → ROONO → RO^•^ + NO_2_^•^ (9)

There are two possible outcomes for ROONO: (1) internal rearrangement with formation of stabler RONO_2_ (Reaction (10)):

RO^•^ + NO_2_^•^ → RONO_2_ (10)

or (2) homolytic break of the O–O bond with the subsequent formation of RO^•^ and NO_2_^•^. The alkoxyl radical can react with NO^•^ (Reaction (11)), which blocks further development of LPO reactions:

RO^•^ + NO^•^ → RNO_2_ (11)

It should also be noted that HNO can reduce the oxoferrylHb form (Hb-Fe^IV^ = O), which is a strong oxidizer itself (Reaction (12)):

porphyrin-Fe^IV^ = O + HNO → porphyrin-Fe^III^ + H_2_O + NO (12)

**Figure 9:**
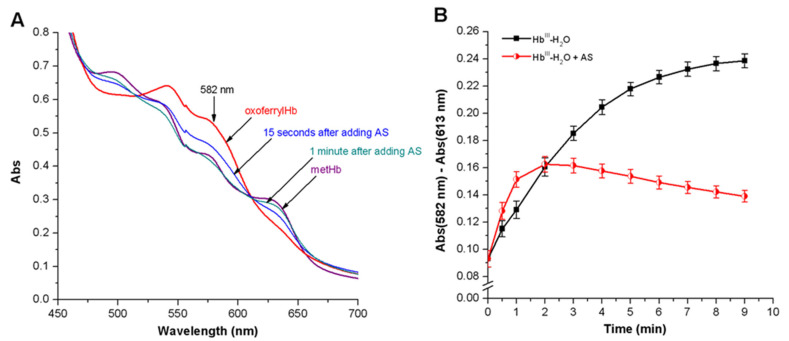
(**A**) Reduction of oxoferrylHb (Hb-Fe^IV^ = O) with Angeli’s salt. Reaction mixture: 0.03 mM Hb in 50 mM PBS (pH 7.4), 0.4 mM H_2_O_2_, 0.12 mM Angeli’s salt. Catalase (0.456 units) was added to remove unreacted H_2_O_2_; (**B**) Effect of Angeli’s salt on formation of oxoferryl-Hb in MetHb-H_2_O reaction with *t*-BOOH. Reaction mixture: 0.03 mM Hb in 50 mM PBS (pH 7.4), 0.71 mM *t*-BOOH, 0.4 mM Angeli’s salt. Results are depicted as the mean with S.E.M. of three independent experiments (*n* = 3). Student *t*-test (**B**) (*p* ≤ 0.05 for all point after 2.5 min).

This HNO effect was confirmed in experiments on Hb-Fe^IV^ = O reduction. OxoferrylHb was obtained with a small H_2_O_2_ excess. Unreacted peroxide was removed with catalase. Adding Angeli’s salt in an equimolar amount led to an almost complete Hb-Fe^IV^ = O reduction to metHb in one minute. This was evidenced by a decrease in optical absorption peaks at λ = 541 and λ = 582 nm, and an increase at λ = 631 nm, which are characteristic of the metHb form (Figure 9A). Angeli’s salt also slowed down Hb-Fe^IV^ = O formation in the Hb oxidation system with *t*-BOOH (Figure 9B).

**Figure 10:**
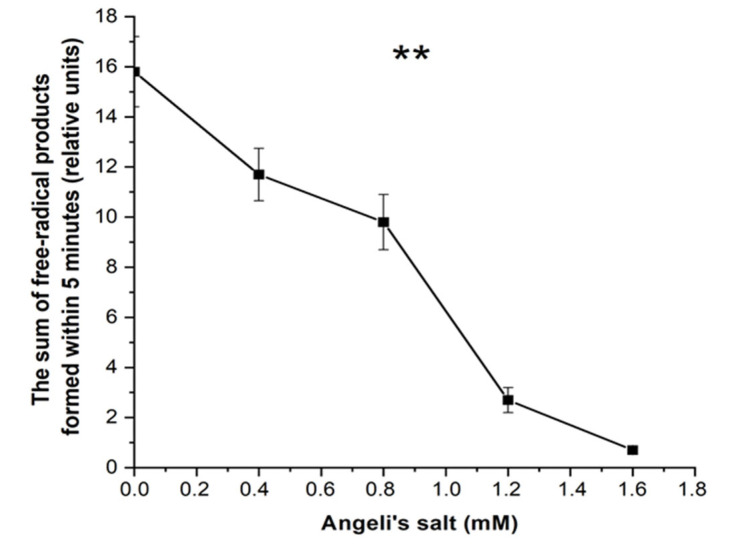
Effect of Angeli’s salt on free radical product formation in the reaction of metHb-H_2_O with *t*-BOOH, measured with *N*^1^,*N*^1^,*N*^4^,*N*^4^-tetramethylbenzene-1,4-diamine (TMPD). Reaction mixture: 0.03 mM Hb, 1.42 mM *t*-BOOH, 0.12 mM TMPD. Results are depicted as the mean with S.E.M. of three independent experiments (*n* = 3). One-was ANOVA (** *p* ≤ 0.1).

Antioxidant activity of HNO was also evaluated based on the effect on TMPD oxidation, measured according to the method described in [31]. Oxidant generation was estimated based on the sum of free radical products formed in the first five minutes of Hb reaction with *t*-BOOH, by calculating the area under TMPD oxidation curve. The NO^−^ anion inhibited release of free radicals in a dose-dependent matter (Figure 10).

## 4. Discussion

AGE formation in vivo and the damage of biomolecules depend not only on the relative concentration of glycating agents (glucose and MG), but also on oxidative conditions [12]. Here, we propose two potential mechanisms for formation of reactive oxygen radicals in case of non-enzymatic glycation. The first mechanism is associated with auto-oxidation of reducing sugar with the subsequent formation of an endiol radical that reduces molecular oxygen with the formation of a superoxide radical and dicarbonyl. This process is called “auto-oxidative glycation”. The second mechanism is glycoxidation. It involves an auto–oxidation of Amadori products with protein dicarbonyls as well as the superoxide radical being formed. Both mechanisms are catalyzed via redox-active transition metal ions [8,44]. Therefore, antioxidants and metal chelators are promising in terms of reducing carbonyl stress effects.

Antioxidants are a separate group of AGE formation inhibitors. There is enough evidence indicating the ability of antioxidants to lower ROS level and to inhibit AGEs formation in vitro and in vivo [34]. Antiglycation compounds with antioxidant activity include polyphenols (caffeic acid, ferulic acid, gallic acid, isoferulic acid, chlorogenic acid, catechin, curcumin, rutin, calycosin) [34,57], alkaloids (berberine, palmatine) [58], terpenoids (oleanolic acid, ursolic acid, betulinic acid), vitamins (A, C, E, B_6_), polysaccharides (from Auricularia and Astragalus) [34], dipeptide carnosine [59,60] as well as derivatives of some natural compounds (N-acetyl-L-cysteine, L-ascorbic acid 2-phosphate) [61]. Antioxidants not only decrease AGE level, but also suppress the intracellular effects caused by AGE action on cell membrane receptors (scavenger receptors and multiligand receptors RAGE) [4]. In addition to capturing free radicals, the main mechanisms of inhibiting AGE formation and functioning are reduction of reactive dicarbonyl compounds, protection of protein structure and AGE decomposition [34].

Our results show that HNO donors can be classified as antiglycating antioxidants. The antiradical and antioxidant effects of HNO were earlier shown. Under oxidative stress, HNO increases the viability of yeast cells deficient in coenzyme Q and inhibits LPO [19] that arises from reduction of free lipid radicals. Moreover, through HNO oxidation, NO is formed, which may also exhibit antioxidant properties and can stop LPO reactions [53,54,55,56,62,63]. HNO can reduce superoxide production both by inhibiting NADH oxidase [64] and by inhibiting mitochondrial respiratory chain complexes I and II [65].

Apart from its direct antioxidant effect, HNO can inhibit non-enzymatic protein glycation by active carbonyl compounds through interaction with protein thiol residues and heme groups [18]. Under physiological conditions, HNO is both an electrophile oxidizing thiols, and a nucleophile that can coordinate and reduce metal ions [66].

Thiols serve as the main biological target for HNO [67]. The rate of this reaction is much higher than the rate of HNO dimerization [12]. HNO reaction with thiols (*k* ~ 10^6^ M^−1^s^−1^) leads to an unstable intermediate being formed, N-hydroxysulfenamide (Reaction (13)). If there is an overage of thiols or vicinal protein thiols, it is converted into disulfide and hydroxylamine (Reaction (14)). N-hydroxysulfenamide can spontaneously isomerize to sulfinamide (Reaction (15)), which is an irreversible thiol modification [18]:

H^+^

HNO + RS^−^ → RSNHO^−^ → RSNHOH (13)

RSNHOH + RSH → RSSR + NH_2_OH (14)

RSNHOH → RS(O)NH_2_ (15)

HNO preferentially reacts with thiolate anions (RS^−^) rather than with a protonated form of thiols (RSH). This means that HNO reactivity to thiols is determined by *pKa* of SH- group and strongly depends on pH [18]. It means that HNO l can act as a site-specific modifier of protein thiol groups. The mechanism of this reaction is the following: nucleophilic sulfur is “attacked” by electrophilic nitrogen from HNO. This is similar to the reaction of thiols with carbonyl compounds, which leads to formation of hemithioacetals [68]. It can be assumed that HNO “competes” with carbonyl compounds for the reactive protein thiols. This may be another explanation for the antiglycation effect of HNO on Hb observed in our study.

Another characteristic of HNO, which is crucial for its biological activity, is its ability to form coordination complexes with metalloproteins, in particular with hemoproteins [69]. HNO reacts with an oxidized heme iron in the reducing nitrosylation reaction (for metMb *k* = 8 × 10^5^ M^−1^s^−1^), forming nitrosylHb (Hb-Fe^II^-NO) (Reaction (16)):

Hb-Fe^III^ + HNO → Hb-Fe^II^-NO + H^+^ (16)

Unlike NO, HNO binds to an oxidized heme iron (*k* > 10^5^ M^−1^s^−1^) much more effectively than to a reduced one (*k* < 10^4^ M^−1^s^−1^) [69].

In our previous work [70], generation of organic free radicals in the reaction of metHb with *t*-BOOH was registered using EPR spectroscopy with a spin trap DEPMPO. We registered nitrosylHb formation, when Angeli’s salt was added to metHb [70]. At the same time, Hb nitrosylation under the impact of HNO inhibited free radical formation almost completely. It is most likely that Hb-Fe^II^-NO does not participate in Reactions (3)–(6); rather, it reduces *t*-BOOH with the formation of nitrite and metHb (Reaction (17)):

porphyrin-Fe^II^-NO + *t*-BOOH + H_2_O → porphyrin-Fe^III^ + *t*-BOH + HNO_2_ + OH^−^ (17)

This mechanism was proposed for the interaction between metmyoglobin and *t*-BOOH [71,72]. In our reaction system, the antioxidant effect of the NO^−^ anion could also be caused by Reactions (16) and (17).

The reaction of HNO with oxyhemes (porphyrin-Fe^II^−O_2_) proceeds with a reaction rate constant of *k* ~10^7^ M^−1^s^−1^ [73,74]. In this reaction, oxidized heme and nitrate are formed (Reaction (18)):

porphyrin-Fe^II^−O_2_ + HNO → porphyrin-Fe^III^ + NO_3_^−^ (18)

It is also possible that, in reaction with oxyhemes, HNO is reduced to H_2_NO and further to NH_2_OH (Reactions (19) and (20)) [73,74]:

porphyrin-Fe^II^−O_2_ + HNO → porphyrin-Fe^III^ + H_2_NO + O_2_ (19)

porphyrin-Fe^II^−O_2_ + H_2_NO → porphyrin- Fe^III^ + NH_2_OH + O_2_ (20)

The antioxidant effect of HNO may also emerge from NO, which is formed either through HNO oxidation by two-electron oxidants (such as flavin adenine dinucleotide), or as a result of reductive nitrosylation of heme proteins (e.g., Hb).

Another potential mechanism of HNO antioxidant effect is associated with coordination iron complexes being formed [75].

Taking into account the fact that membranes are a favorable environment for HNO [76], it could be assumed that HNO provides the most effective protection against oxidative damage in case of membrane proteins and lipids. The inhibition of LPO is another mechanism for antiglycation effect of HNOa, since reactive dicarbonyl compounds that additionally generate AGEs can be produced during LPO reactions.

Angeli’s salt and Piloty’s acid were used as effective treatments for cardiovascular diseases and cancer [24]. Their application as cardioprotective agents is especially promising, since they have a hypotensive effect, are able to improve myocardial contractility and inhibit its hypertrophy [77,78,79]. Resistance to HNO donors is not developed [78,80], unlike in the case of organic nitrates actively used for vasodilation as NO donors. The reason for this could be that HNO and NO^−^ do not react with superoxide, whereas NO is rapidly utilized in reaction with this anion radical.

Combining HNO cardio- and vasoprotective effects with antioxidant and anti-glycation properties may be promising for the development of pharmaceutical preparations with a synergistic therapeutic effect. These putative therapeutics could be used to protect the cardiovascular system and nervous tissue cells in the case of diseases associated with oxidative and carbonyl stress.

## 5. Conclusions

This study showed that inhibitors of AGE formation can be developed on the basis of HNO donor, Angeli’s salt, which is an HNO-based AGE formation inhibitor. It was also shown that HNO can “quench” reactive radical intermediates formed both under carbonyl stress and during LPO. Antioxidant and antiradical HNO properties are related to being a donor of both hydrogen atom and NO molecule.

**Figure 11:**
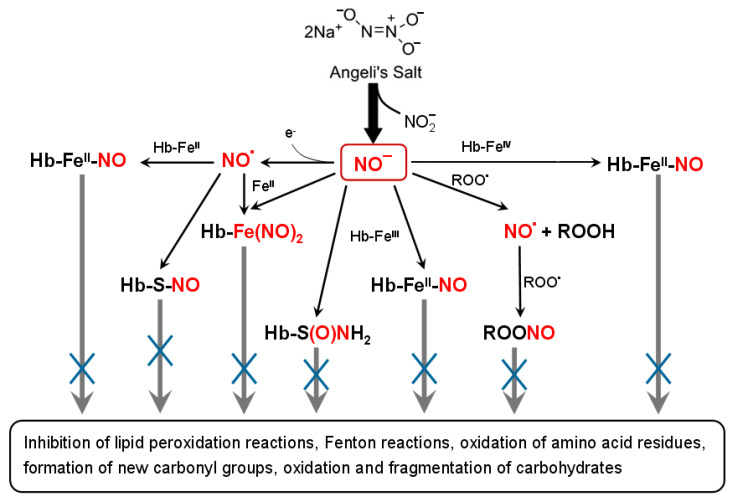
Schematic representation of antioxidant and antiglycation effect of HNO donor, Angeli’s salt, on hemoglobin.

HNO retarded MG-caused Hb modification during the non-enzymatic glycation reaction. This is especially important since MG action largely contributes to the carbonyl stress, acting, among them, on erythrocytes and Hb [27,81]. The antiglycation effect of HNO is due to its ability to intercept free radicals that arise during the non-enzymatic glycation and oxidation reactions of Hb. It can be also connected with HNO ability to ensure metHb nitrosylation and oxoferrylHb reduction. In Hb reaction with *t*-BOOH, HNO slowed down the decomposition of the heme group, formation of carbonyl derivatives and inter-subunit crosslinking, and decreased the amount of free radical products. Therefore, our results demonstrate that HNO donor Angeli’s salt can decrease oxidative stress and reverse the stress effect on biomolecules caused by non-enzymatic glycation (Figure 11).

## Data Availability

The data presented in this study are available in the article.
